# Unobtrusive occupancy and vital signs sensing for human building interactive systems

**DOI:** 10.1038/s41598-023-27425-6

**Published:** 2023-01-18

**Authors:** Chenyan Song, Amy D. Droitcour, Shekh M. M. Islam, Avon Whitworth, Victor M. Lubecke, Olga Boric-Lubecke

**Affiliations:** 1 Adnoviv, Inc., Honolulu, HI 96822 USA; 2 Department of Electrical and Electronic Engineering, University of Dhaka, Dhaka, 1000 Bangladesh; 3 Department of Electrical and Computer Engineering, University of Hawaii at Manoa, Honolulu, HI 96822 USA

**Keywords:** Energy infrastructure, Electrical and electronic engineering, Biomedical engineering

## Abstract

Cognitive buildings use data on how occupants respond to the built environment to proactively make occupant-centric adjustments to lighting, temperature, ventilation, and other environmental parameters. However, sensors that unobtrusively and ubiquitously measure occupant responses are lacking. Here we show that Doppler-radar based sensors, which can sense small physiological motions, provide accurate occupancy detection and estimation of vital signs in challenging, realistic circumstances. Occupancy was differentiated from an empty room over 93% of the time in a 3.4 m × 8.5 m conference room with a single sensor in both wall and ceiling-mounted configurations. Occupancy was successfully detected while an occupant was under the table, visibly blocked from the sensor, a scenario where infrared, ultrasound, and video-based occupancy sensors would fail. Heart and respiratory rates were detected in all seats in the conference room with a single ceiling-mounted sensor. The occupancy sensor can be used to control HVAC and lighting with a short, 1–2 min delay and to provide information for space utilization optimization. Heart and respiratory rate sensing could provide additional feedback to future human-building interactive systems that use vital signs to determine how occupant comfort and wellness is changing with time.

## Introduction

An occupant-centric approach to building management requires sensing how occupants use and interact with their environment and using that data to proactively adjust the environment to improve occupant comfort, optimize space utilization, and reduce energy consumption^1^. Wearable sensors that measure skin temperature, respiration rate, and heart rate have been proposed to provide information on vital signs and changes in vital signs to provide data about human responses to enable more effective human building interactions^2,3^. A limitation of using wearable sensors is that it relies on occupants using the devices and opting to share that data with the building management system. Non-intrusive infrared thermography^4,5^ and optical imaging^6^ have been proposed as alternative non-contact methods to evaluate thermal comfort. These methods provide an indirect assessment of skin temperature, which is a limited measure of overall physiological response to the built environment. Real-time visual perception using human eye pupil size measurements has been proposed to evaluate human response to lighting parameters^7^. However, pupil size measurement requires interaction with a computer screen and does not provide feedback on other parameters. Innovative sensing, data analytics, algorithms, and tools are required to make Human Building Interaction (HBI) a reality. It remains a challenge to estimate the comfort and response of all occupants unobtrusively and ubiquitously.

Occupancy and vital signs sensors may help to create healthy occupant experiences and provide sustainable solutions for the built environment. Following COVID shutdowns, people are returning to offices with more awareness of the importance of environmental conditions to employee health and productivity. Companies are more aware of the potential cost savings from reduction in real-estate footprint, which can be facilitated by room-level occupancy data^8^. Employees need positive workplace experiences and respectful safety and security to be happy and productive in working and collaborating in offices^9,10^; buildings that sense occupant needs and proactively adjust lighting and ventilation can meet those demands. Occupancy sensors can provide HBI systems with data required to optimize space utilization. In the future, occupant vital signs and changes in these parameters may be used with other sensors to provide information about wellness and comfort of occupants. Information about occupancy and comfort may enable proactive adjustment of lighting, temperature, and ventilation to maintain occupant wellness, comfort, and productivity while reducing energy consumption^8–10^.

Motion-sensing occupancy sensors, such as those using passive infrared (PIR) and ultrasound (US), are popular as a means to control lighting to save energy, although they have significant failure rates when occupants are sedentary, which leads to setting long delay periods, during which rooms are assumed to be occupied following movement, diminishing accuracy of occupancy information and its energy saving potential^11^. Carbon dioxide sensors are sometimes used to infer occupancy and even the number of occupants, but there is a considerable lag between occupancy and increases in carbon dioxide levels^12^. Image sensors tend to be more accurate, but many occupants are uncomfortable with the potential privacy implications of cameras in every room^13^. Arrayed occupancy sensor technologies such as floor pressure pads^14^ and time of flight infrared^15^ measurements have significant installation challenges and costs, making them unlikely choices for retrofit applications. Doppler radar sensors can provide room-level occupancy with high accuracy for sedentary occupants, without long lag times, without introducing privacy concerns, and in a low-cost, easy-to-install form factor, while also providing the potential for non-contact vital signs sensing.

## Literature review

The research published in recent years on occupancy sensing using Doppler radar indicates that activity and physiological sensing could be used to remotely evaluate occupant response to environmental conditions, which would lead to major changes in HBI. Gennarelli et al.^16^ achieve an occupancy/vacancy accuracy of greater than 96% by applying standard deviation, histogram and Doppler spectrum energy methods to a continuous wave quadrature Doppler radar for real-time occupancy sensing in indoor environments. Lurz et al.^17^ emulate human respiration using a metallic plate mounted on a linear stage and demonstrate a human subject at null point still can be detected with a single channel low-power 24 GHz receiver. The work in^18^ exhibits the feasibility of utilizing Doppler radar sensor (RFBeam K-LC3) and Infrared Thermal Array Sensors with a Deep Neural Network (DNN) model to build an effective occupancy detection framework. In^19,20^, ranging Doppler radar systems using 60 GHz and 24 GHz Infineon Radar chip sets (BGT60TR24B and BGT24MTR11, respectively) are used to detect the occupants’ presence and count occupant number for up to 3 occupants in a typical office environment. In addition to active radar systems, passive radar receivers have been used for occupancy detection and activity recognition by harvesting RF signals in the environment. Examples include a Passive Wi- Fi Radar (PWR) technique for occupancy detection and people counting in^21,22^ and a Passive IoT Radar (PIoTR) system that uses RF transmissions from IoT devices for human monitoring in^23^.

**Figure 1:**
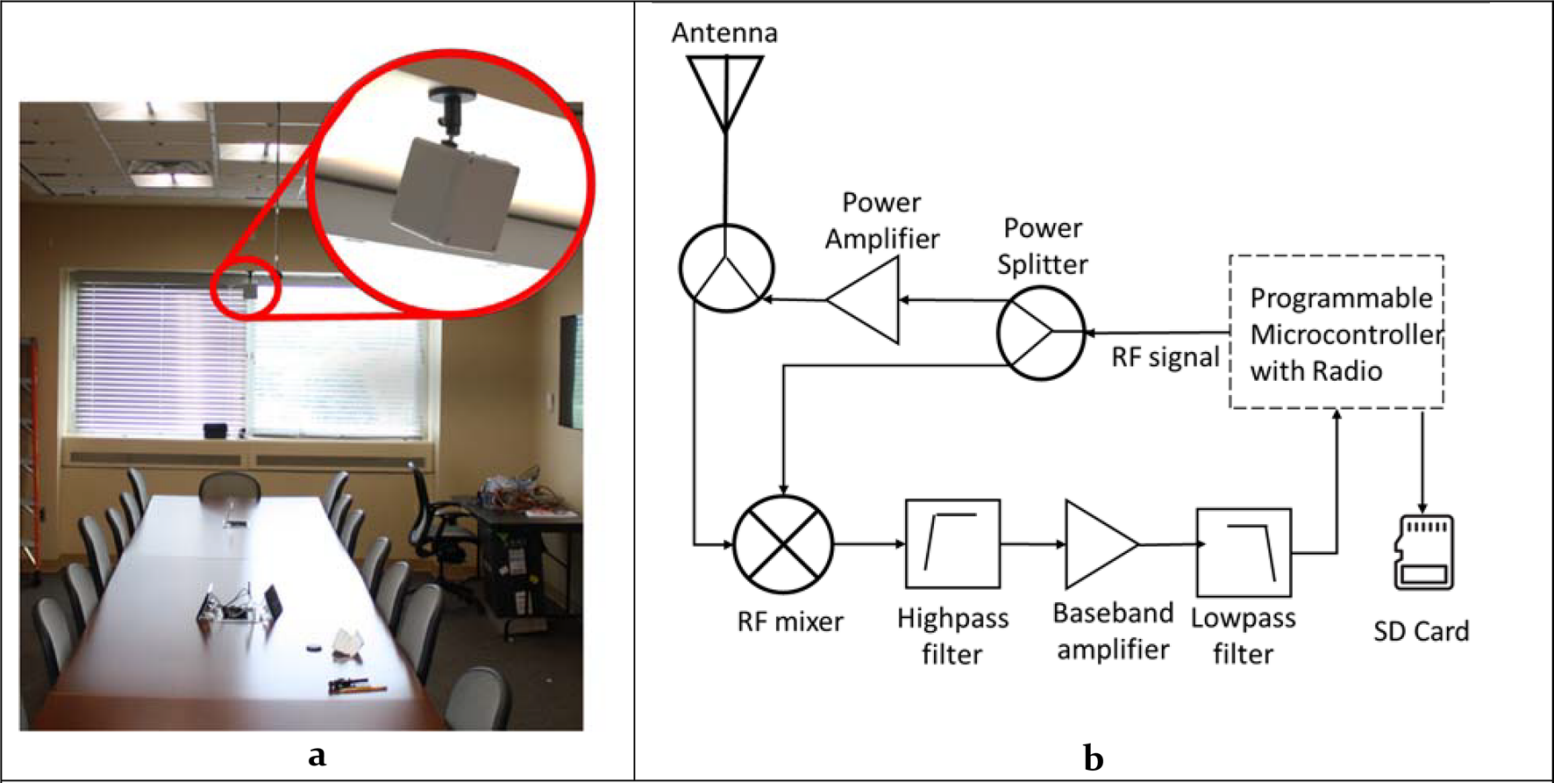
(**a**) The 3.4 m × 8.5 m LESA Smart Conference Room, with the TruePODS sensor mounted above the window on the wall—this is the “wall-mounted” configuration. The TruePODS sensor enclosure dimensions are 12 cm × 12 cm × 10 cm. Photographs originally taken by Michelle Simkulet, and modified with permission by the authors. (**b**) Block diagram of the TruePODS radar transceiver. An RF signal is generated on the programmable microcontroller and split into two signals for transmitting and downconversion. Recorded data is output to an SD card for further analysis.

While the progress in works cited above is notable, these works have some limitations, such as short experimental duration (up to 60 s for^16–20^), the inferior sensitivity of passive radar systems for detecting stationary persons^21,22,23^, and detection of stationary persons only at 2–3 m from the sensor^16,17^. The True Presence Occupancy Detection Sensor (TruePODS™) presented in this paper is a Doppler radar-sensor that detects physiological motion to indicate whether a space is occupied or vacant^24^. With additional algorithms, this sensor can be used to provide occupant vital signs over time, as well as occupant count. It has been developed by a start-up company Adnoviv in collaboration with the University of Hawaii building on previously published work^24–28^. Compared with previous works published by the authors and the work demonstrated by other groups mentioned above, this work presents the first fully integrated prototype using simple, low-cost single channel radar that has been validated under realistic conditions (Fig. 1a).

The TruePODS was tested in the LESA Smart Conference Room, shown in Fig. 1a, and the data was analyzed to determine accuracy of occupancy/vacancy estimation with different algorithms, and to assess accuracy of estimated heart and respiratory rates. Occupancy and vacancy were accurately differentiated over 93% of the time, using two-minute or shorter data segments, and using both wall and ceiling-mounted configurations, selecting challenging data sets where occupants were mostly stationary. Heart and respiratory rates were accurately detected in all seats in the conference room with a single ceiling-mounted sensor.

## Method and materials

### TruePODS occupancy and vital signs sensor

The TruePODS radar transceiver transmits an electromagnetic signal. When this signal hits objects in the room, it reflects with a phase shift that is proportional to the object’s distance, and over time, this phase shift is proportional to the objects’ motion. If a stationary person is present, the phase shift of the reflected signal is proportional to the tiny movement of the chest surface due to cardiorespiratory activity. This reflected signal is received by the radar, where it is downconverted so the phase of the signal can be demodulated. This phase signal is proportional to the physiological motion, which is then used with a detection algorithm to determine occupancy/vacancy, and an estimation algorithm to determine heart and respiratory rates^29^.

The TruePODS is a custom homodyne Doppler radar, with a block diagram shown in Fig. 1b. The hardware includes a programmable microcontroller with a built-in 2.4-GHz ISM band radio, two RF power splitters and a power amplifier, a single custom airgap patch antenna with a 60° beamwidth that is used for both transmitting and receiving, and signal-conditioning electronics that filter and amplify the baseband signal prior to digitization. A continuous wave radio signal in the 2.4 GHz ISM band is generated by the RF signal source on the programmable microcontroller. This RF signal is split in two with a power splitter so it can be used for both transmission and downconversion. The transmit portion of the signal is amplified and fed to the antenna through other power splitter, and the local oscillator signal for downconversion is fed to the RF mixer’s local oscillator input port. The signal is transmitted by the antenna, and the reflected signal is received by the same antenna. The power splitter directs the received signal to the mixer’s RF input port. The mixer downconverts the signal to baseband, and its output is a baseband signal proportional to the motion of the objects in the room. This baseband signal is amplified and filtered before being digitized. Although the processor can perform real-time processing, in this work the digitized baseband data was stored on an SD card for subsequent processing and analysis. The radio can be programmed to operate between 2.4 and 2.5 GHz, and in this work it was programmed to operate at 2.4 GHz, and the transmit power at the antenna port was about 16 dBm (40 mW). The radio and antenna were placed inside a plastic enclosure that measures 12 cm × 12 cm × 10 cm, as shown in Fig. 1a. The sensor was powered via wall power with a USB adapter.

### Validation testing in smart conference room testbed

TruePODS modules have been tested in the Smart Conference Room testbed in the LESA (Light Enabled Systems & Applications) Center at Rensselaer Polytechnic Institute. The Smart Conference Room (SCR), shown in Fig. 1a, has dimensions of 3.4 m × 8.5 m, seats up to 23 people, has occupancy and lighting sensors, and color changeable lighting^30^. Infrared time-of-flight sensors detect occupant location and pose (sitting, falling, standing). A mesh network of color sensors provides coarse occupancy sensing and measures reflected sunlight and solar heat flux^31^. Over 27 h of data has been collected with the TruePODS system in the SCR, covering a number of variables, which are outlined in Table 1. Data was collected with an empty room on each day that the sensor was tested. Data has been collected with a single person sitting quietly in all the different seats at the conference table and in the corners of the room, and with a single person moving with fine motion (typing, fidgeting, or swiping on a phone), and with larger motion such as walking or gesticulating. Data has also been collected with 2–10 people sitting quietly, with fine motion, and with large motion in different positions in the room. Data has been collected in controlled settings and during regular meetings that occur in the conference room. These measurements involved human subjects, and as such were performed in accordance with a protocol approved by the Rensselaer Polytechnic Institute Internal Review Board (protocol ID#2023). All research was performed in accordance with the protocol and with relevant guidelines and regulations, and all test subjects gave informed consent.

**Table 1:** Variables in Smart Conference Room data collection, with the amount of time each option was used rounded to the nearest quarter hour.

Mounting	Wall mounting	Ceiling mounting	
	4.25 h	22.75 h	
Occupancy	Empty	Single occupant	Multiple occupants
	9.25 h	6.75 h	11 h
Reference	Video	Time of flight	
	22 h	6.5 h	
Vital signs	Breathing normally, HR not noted	Breathing in Sync	HR recorded
	22 h	2.5 h	2.5 h

### Occupancy/vacancy detection algorithms

The data obtained with an empty room and with primarily stationary occupants were analyzed to evaluate the detection accuracy of the TruePODS sensor. The TruePODS can easily discern the signals produced by human locomotion and fidgeting, so the algorithm testing focused on the most challenging cases of stationary occupants, where the only motion is breathing.

To determine the occupancy status of the conference room with the data collected via TruePODS, algorithms based on Riemann Integral (RI) and Band Power (BP) were developed in MATLAB^32–34^. In both the RI and BP algorithms, a sliding window method was used, i.e., the data in each recorded file were divided into continuous windows of a specified length. Each window contains samples overlapped with the previous window. The signals detected and recorded by TruePODS contain DC offsets that are induced by reflections from static objects; these DC offsets are removed by subtracting the mean of the raw data in each window from each sample. Since respiration signals are below 1 Hz, a low-pass filter is also employed in each window to remove high-frequency noise. After the signals are windowed, filtered, and have their DC offset removed, these conditioned signals are analyzed with either the RI or the BP method, as described below.

#### RI method

In each window, the RI algorithm integrates all the samples and compares the result with a preset threshold. The following equation shows the calculation of RI in the k-th sliding window of the conditioned radar signal *x(t)* as:

1 $$RI(k)= \sum_{n=0}^{N-1}\left|{x}_{k}(n)\right|$$

where N denotes the total number of samples in each window. The RI in each window is compared to a pre-set threshold to determine occupancy; if the RI is above the threshold, the room is considered occupied, and if the RI is below the threshold, the room is considered vacant. The RI threshold is calculated from data recorded in an empty room as the average value plus 1.5 times the standard deviation.

#### BP method

In each window, the BP algorithm calculates the average power in a specified frequency band corresponding to a typical range of respiration rates and compares that value to a pre-set threshold. In this work, the frequency band used was 0.1–0.3 Hz, corresponding to a wide range of adult resting respiration rates of 6–18 breaths per minute. The average power in the specified respiratory frequency range was determined by the Matlab *bandpower* function, which applies a modified periodogram to the signal. The BP threshold is calculated as the average power plus 1.5 times the standard deviation from data recorded when the room is empty. If the BP value exceeds this threshold, the room is considered occupied, and if it is below the threshold, the room is considered vacant.

### Vital signs estimation

In this analysis, respiratory and heart rates were determined by segmenting the signal to remove sections with high amplitude (indicating large motion), and then analyzing segments that were 10–30 s long. This segment is long enough to typically include at least three respiration cycles. To estimate respiration rate, the segmented signals were filtered with a Finite Impulse Response (FIR) bandpass filter with corner frequencies at 0.05 Hz and 1 Hz to remove out-of-band noise and DC offsets. To estimate heart rate, the segmented signals were filtered with a FIR bandpass filter with corner frequencies at 0.8 Hz and 3 Hz to remove respiration and out-of-band noise, while still digitizing the fundamental and the second harmonic of the heart signal. Adult resting heart rates are typically 50–90 beats per minute, or 0.83–1.5 Hz, so the second harmonics are at 1.67–3 Hz. The Fast Fourier Transform (FFT) was applied to the filtered signals to generate the signals’ frequency spectra. The frequency associated with the maximum amplitude of the FFT was converted to breaths per minute to estimate the respiratory rate and to beats per minute to estimate the heart rate. This process is represented with experimental data in Fig. 4.

To determine the accuracy of vital signs estimation, in some measurements, occupants were instructed to breathe at a specific frequency: 8, 10, 12, 15, or 18 breaths/min, and heart rate was periodically recorded from a Fitbit Charge 4 Smart Watch.

## Results

### Occupancy/vacancy sensing

The detection accuracy of the RI and BP methods was quantified as the amount of time vacancy or occupancy was **correctly** detected by TruePODS using each method divided by the **total** time measured, converted to a percentage. The higher the percentage, the more accurate the method is. The **true** vacant or occupied time was determined by the video reference data for most measurements except for the cases where video was not recorded (to preserve privacy during meetings) and the LESA infrared time-of-flight sensor data were used for reference.

With the 100 Hz sampling rate of TruePODS, to achieve 0.1 Hz resolution, a minimum window size of 10 s or 1000 samples is required for the BP calculation. However, when analyzing the data, we found the selection of the window size impacts the accuracy of the method. This may be because respiration is not a constant activity—it varies with time—and a longer window will capture more variation. After studying the impact of various window sizes on the detection accuracy of occupancy status for both BP and RI methods, a 1-min window with 30 s overlap for wall-mounted TruePODS and 2-min window with 1-min overlap for ceiling mounted TruePODS were determined to produce the best accuracy when using BP method, while 1-min window with 30 s overlap was optimum for both installation configurations when using the RI method.

Table 2 summarizes the detection accuracies of wall-mounted and ceiling-mounted TruePODS sensors using BP and RI methods to detect and determine the vacancy/occupancy of the room. To evaluate and assess the accuracy of TruePODS and the algorithms, data were collected for a various length of time over several days. The day-by-day accuracy of TruePODS was determined by comparing the detection results with the video or infrared reference as described above. The overall accuracy was determined by weighted mean of the determined accuracies as shown by following equation:

2 $$Overall Accuracy \left(\%\right)=\frac{\sum_{i=1}^{n}{x}_{i} \times {w}_{i}}{{\sum }_{i=1}^{n}{w}_{i}},$$

where *n* is the number of testing days, *w*_*i*_ is the testing time for each day, *x*_*i*_ is the accuracy for each day. The standard variation of the weighted mean accuracy was calculated in Eq. (3) as follows:

3 $$STD=\sqrt{\frac{{\sum }_{i=1}^{n}{w}_{i}{\left({x}_{i}-\overline{x }\right)}^{2}}{\frac{n-1}{n}{\sum }_{i=1}^{n}{w}_{i}}},$$

where *n* is the number of testing days, *w*_*i*_ is the testing time for each day, *x*_*i*_ is the accuracy for each day, $\overline{x}$ is the weight mean accuracy determined by Eq. (2).

**Table 2:** Detection accuracy summary of wall-mounted and ceiling-mounted TruePODS sensor.

Wall mounted TruePODS				Ceiling mounted TruePODS			
Date	Test time (min)	BP accuracy (%)	RI accuracy (%)	Date	Test time (min)	BP accuracy (%)	RI accuracy (%)
6/11/21	35	94.29	92.86	7/23/21	65	95.38	97.62
6/14/21	28	98.21	92.86	7/29/21	51	92.16	93.00
6/17/21	45	90.00	92.22	8/3/21	56	89.29	86.96
6/18/21	36	94.44	84.72	8/9/21	52	94.23	89.11
6/21/21	14	96.43	85.71	8/28/21	16	93.75	88.24
6/22/21	13	92.31	92.31	8/30/21	16	87.50	87.50
6/28/21	23	91.30	91.30	9/3/21	51	98.04	97.12
7/9/21	64	96.09	96.09				
Overall	258	94.19	91.86	Overall	307	93.49	92.32
STD	–	2.64	3.74		–	3.12	4.37

**Figure 2:**
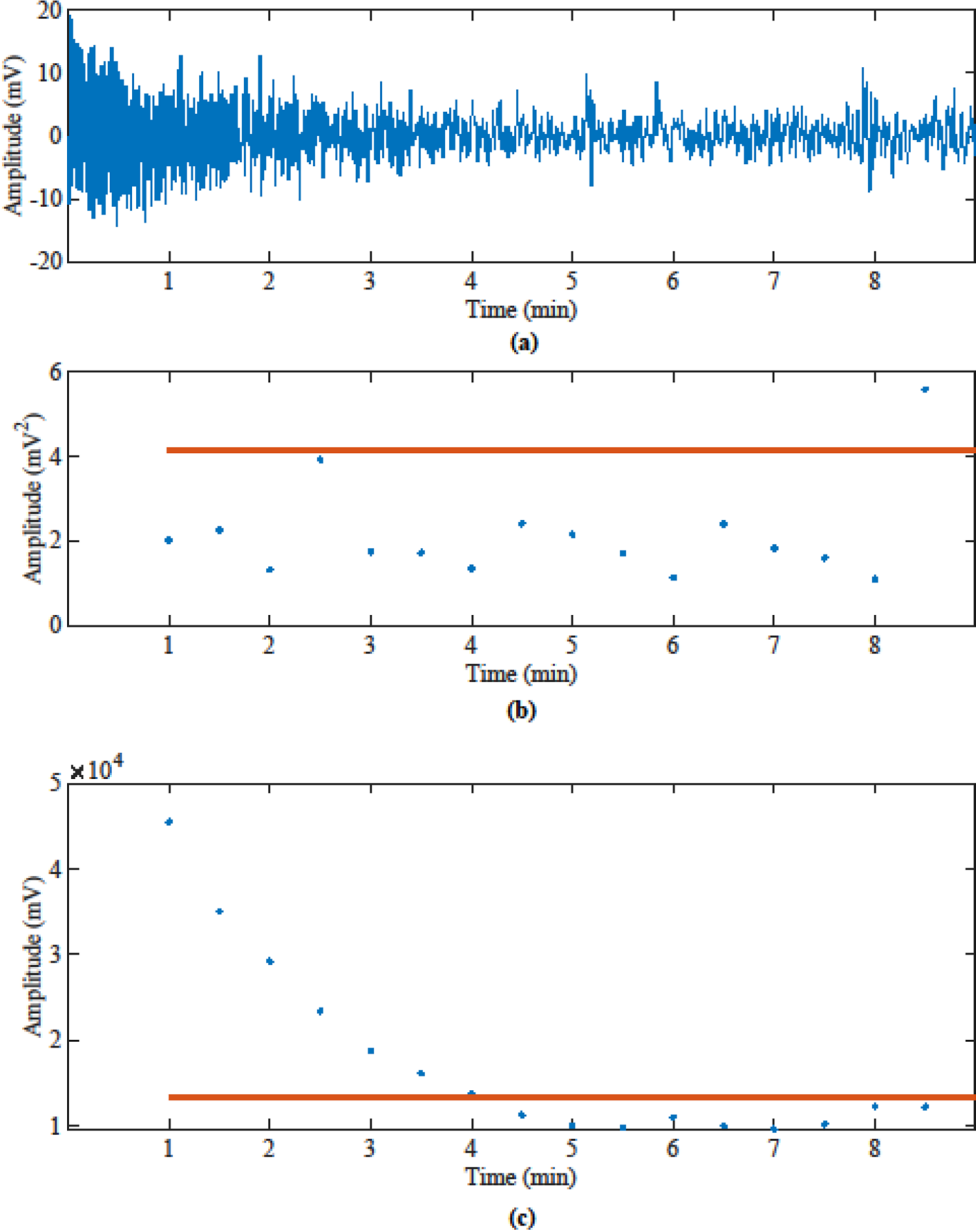
Vacancy detection of wall mounted TruePODS sensor for an empty room, using 1-min sliding-window with 30 s overlapping: (**a**) filtered radar signals after static-background being removed still contain a lot of noise outside the respiration band; (**b**) most of the noise is correctly discerned by BP method, which shows the room as vacant (blue dots below the red line indicate vacancy); (**c**) RI method cannot discern the large amplitude signal in the empty room potentially caused by vibration after plugging in the sensor and it reports false occupied signals for the first 4 min (where blue dots are above the red line) before correctly indicating vacancy (when blue dots drop below the red line).

The results in Table 2 show that the TruePODS sensor using the BP method can achieve day-by-day accuracies of above 87% and about 94% of overall accuracy with variation of about 3% when occupants are primarily sedentary, or the room is vacant (accuracy would be higher were data sets with high numbers of occupants and primarily moving occupants included). This is slightly better than the RI method, which has day-by-day accuracies of above 85% and about 92% of overall accuracy with variation of about 4%. The BP method is likely more accurate because it focuses on the power in the respiration frequency band, and most noise or interference is outside this band, while noise or interference can still trigger the RI threshold. This is illustrated in Fig. 2, which includes data collected when the smart conference room was empty. Damped oscillations appear in the first 4 min of the radar signal detected by the TruePODS. Since the oscillations of the data are not within the respiration frequency range, BP method provides 95% detection accuracy, much better than RI method which reports the room as occupied for almost half of duration of this data set.

**Figure 3:**
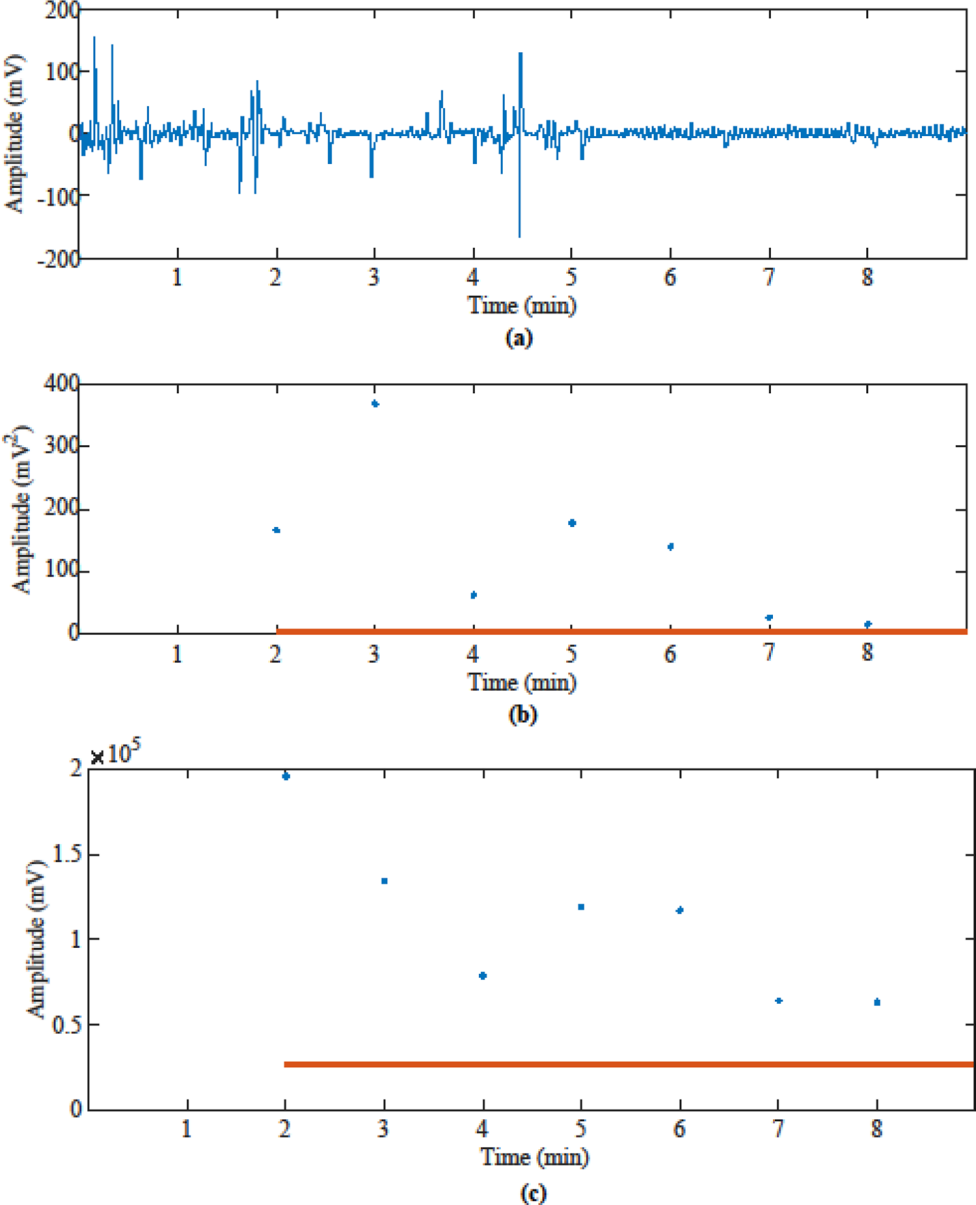
Ceiling Mounted TruePODS sensor detects a person hiding under table: (**a**) filtered radar signals after static-background being removed; (**b**) band power results (blue dots) are consistently above the band power threshold (red line), so occupancy was accurately detected 100% of the time using the BP algorithm, (**c**) Riemann Integral results (blue dots) are consistently above the RI threshold (red line), so occupancy was accurately detected 100% of the time using the RI algorithm.

Figure 3 shows an example of detection results for the ceiling-mounted TruePODS sensor, using the two methods described above to determine the occupancy when a person lay under the table in the Smart Conference Room. This is a particularly challenging case where the subject is not optically visible from the sensor and cannot be detected by infrared or ultrasonic occupancy sensors, but can potentially be detected with the TruePODS. The video reference showed that the occupant got under the table first, then adjusted their posture and fidgeted for a few minutes, and finally lay down on the floor and kept this still posture for the rest of this testing period. These motions were clearly detected by the TruePODS sensor, as shown in Fig. 3a where the DC-removed and filtered radar signals are shown. The first 5 min of the data demonstrates signals of large and small amplitude which indicate locomotion and fidgeting, which can be discerned by the TruePODS sensor easily. The rest of the data are small amplitude periodic signals that indicate detection of respiratory motion while the occupant was lying completely still. Figure 3b shows that the calculated power in the specified frequency band of 0.1–0.3 Hz for each window (blue dots) is above the preset BP threshold (red line). Figure 3c shows that the calculated RI for each window (blue dots) are above the preset RI threshold (red line). In this testing, a 2-min window was updated each minute (except the first window was 2 min after the test started to fill the window). Both BP and RI methods determined the room is occupied when the occupant was under the table with 100% detection accuracy compared with the video reference, while the time-of-flight infrared sensor could not detect the occupant under the table.

### Vital signs estimation

Vital signs estimation was evaluated for data in which a single occupant was sitting still and a respiratory rate and/or heart rate was known. For respiratory rate, the known rate was obtained by instructing the occupant to breathe at a consistent rate (selected by the subject) in accordance with a metronome. For heart rate, the known rate was obtained by recording the heart rate displayed on a smart watch every few minutes. If the respiratory rate obtained was within 1 breath per minute of the metronome rate, it was considered to be accurately detected. If there was no respiration reference for a location that had a TruePODS measurement, and the respiration signal was clearly visible and provided a rate in the typical respiration band, it was also considered to be detected. If the heart rate obtained was between the highest and lowest heart rates recorded during the measurement period, it was considered to be accurately detected.

In the initial experiments, the TruePODS sensor was mounted near the top of the wall, as shown in Fig. 1a. An occupant was breathing in accordance with a metronome at a selection of seats around the table, and in each corner of the room. For the wall-mounted sensor, respiratory rate was detected in every seat at the table at which it was measured, and in three corners of the room, including the two far corners which were over 9 m from the sensor. In the corner where respiratory rate was not detected, presence was detected from fine motion. Heart rates were not recorded while the TruePODS sensor was wall-mounted, so heart rate detection was not analyzed for the wall-mounted scenario.

In the next set of experiments, the sensor was mounted on the ceiling in the center of the room, above the table. For these experiments, an occupant sat at all seats at the table and stood in each corner of the room, and in various experiments, breathing was in accordance with a metronome and heart rates were periodically recorded from a smart watch. With the ceiling-mounted sensor, respiratory rates and heart rates were accurately detected in all positions that were tested. The corners were 5 m from the sensor, which was located on the ceiling in the center of the room.

These data were further analyzed to quantify the accuracy of heart and respiratory rates. For respiratory rate analysis, the difference between the TruePODS rate and rate at which the occupant was instructed to breathe was calculated, and the percentage of the reference rate was also calculated. Note that occupants do not always perfectly breathe at the instructed rate, even while using a metronome. In Table 3, the mean and standard deviation of the difference is shown for the 6 wall-mounted respiration measurements and for the 21 ceiling-mounted respiration rate measurements. The mean difference was 1.3% of the instructed rate for wall-mounted and 3% of the instructed rate for ceiling mounted, with standard deviations of 1.5% and 12.6%, respectively.

**Table 3:** Analysis of respiratory rate estimation accuracy.

Mounting	Number of measurements	Respiratory rate: difference between metronome rate and rate measured with TruePODS			
		Mean of difference(resp/min)	Std deviation of difference (resp/min)	Mean % difference (%)	Std. deviation of % difference (%)
Wall	6	0.2	0.2	1.3	1.5
Ceiling	21	0.2	1.4	3.0	12.6

The difference between the TruePODS heart rate and the reference heart rate was also measured. This analysis considers the difference between the heart rates measured by the TruePODS, and the closest in time measurement of heart rate recorded manually by the occupant from the Fitbit Charge 4 smart watch. Data is divided into 3 categories based on how the reference was recorded, as shown in Table 4. The first data set is where the occupant sat in every seat at the table for 2 min per seat and recorded the smart watch heart rate in the middle of the 2-min interval. The mean difference was 0.1 beats/min, and the standard deviation of the difference was 4.1 beats/min. The second set is data where the occupant sat at a handful of seats at the table and stood in 2 corners for 5 min per location, recording the smart watch heart rate at the beginning, middle, and end of the time in that location. The third data set had the heart rate was recorded every 5 min, and the occupant changed seats every 2 min. The mean difference in this dataset was − 2.4 beats/min and the standard deviation of the difference was 9.6 beats/min. These differences that are within 10% are similar to the accuracy numbers of the Fitbit Charge 4 when compared with a Holter monitor^35^.

**Table 4:** Analysis of heart rate estimation accuracy.

Reference measure frequency	Number of measurements	Heart rate: difference between smart watch rate and rate measured with TruePODS			
		Mean of difference (beats/min)	Std deviation of difference (beats/min)	Mean % difference (%)	Std. deviation of % difference (%)
Mid-2 min measurement	18 (one per seat)	0.1	4.1	0	6
3 times per 5 min measurement	6	− 7.5	5.1	− 11	8
Periodically (every 5 min, with seat changes every 2 min)	24 (includes many seats and all 4 corners)	− 2.4	9.6	− 4	12

**Figure 4:**
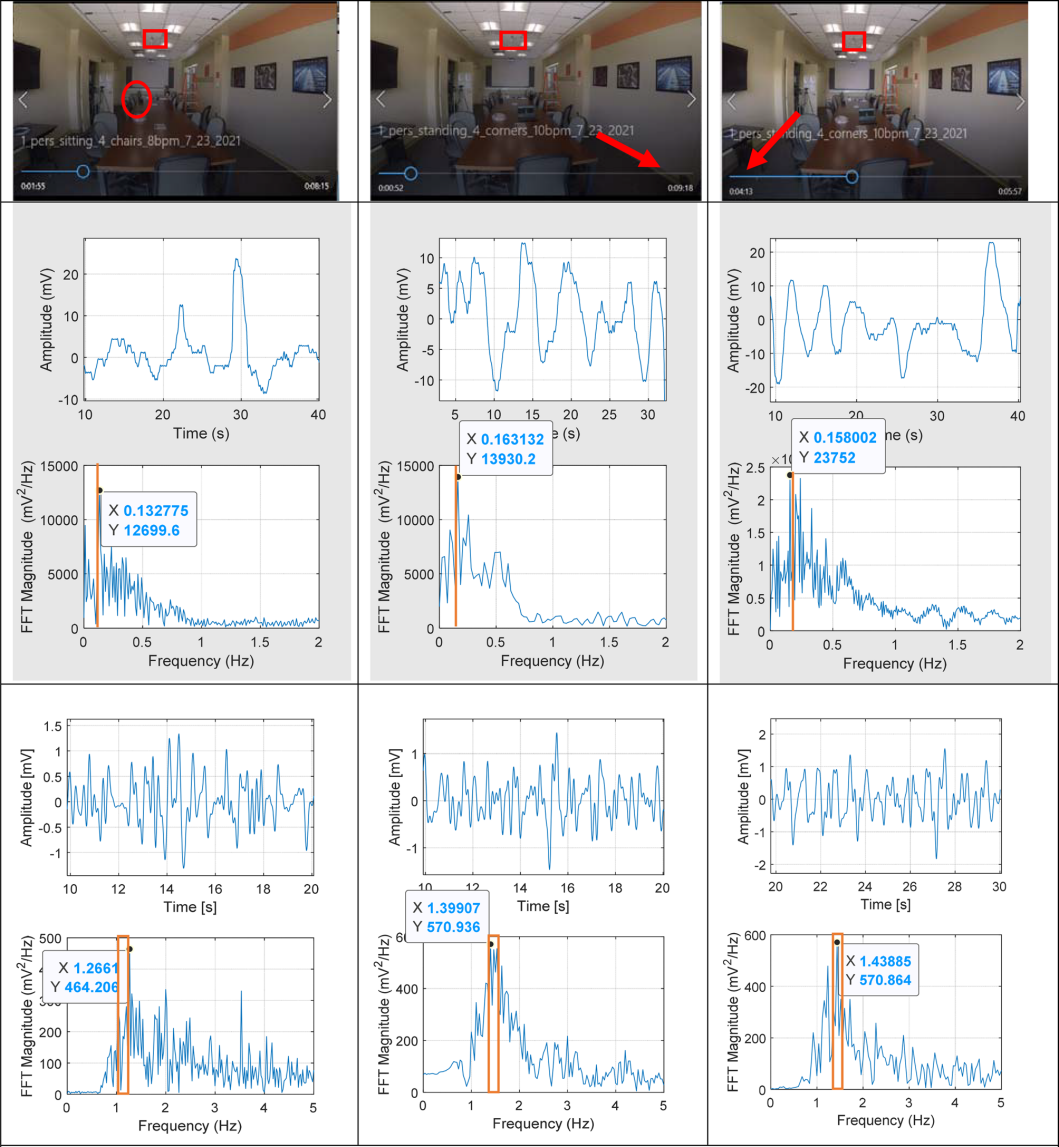
Physiological data examples from an occupant at three locations: the far corner of the table, and the two near corners; each column shows a different location. The top is a picture of the room, with the sensor outlined with a red box, and the occupant location circled if in the image, or an arrow to the location if out of the image. Below the picture is a sample of a signal filtered for respiratory motion: the top is a 30-s segment in the time domain, and below that is the Fast Fourier Transform (FFT) of the signal, showing it in frequency domain, with the rate at which the subject was instructed to breathe indicated with an orange line. At the bottom there are samples of signals filtered for heartbeat: the top is a 10-s segment in the time domain, and below that is the FFT of this signal, showing it in the frequency domain, with the range of rates recorded from a smart watch indicated by an orange box. (Photographs originally taken by Michelle Simkulet, and modified with permission by the authors.).

A sample of heart and respiration signals that were detected from occupants in different locations in the smart conference room, while the TruePODS sensor is mounted on the ceiling in the center of the room, are shown in Fig. 4. In all three shown cases, the heart rate detected via TruePODS closely matches that recorded by the occupant from a smart watch and the respiratory rate estimated by the TruePODS matches the rate which the occupant chose and maintained with the help of a metronome. At the top, still images of the room during the measurements are shown, with the sensor outlined with a red box, and the occupant identified with a red circle if in the image or pointed to with a red arrow if out of the image. On the left the occupant is at the far corner of the table. In the center the occupant is in the near right corner of the room, and on the right the occupant is in the near left corner of the room. Below the still images are 30-s samples of signals from each location filtered to identify respiration with a 4 Hz lowpass filter, shown in the time domain and frequency domain. At the bottom, 10-s samples of signals from each location, filtered to identify the heartbeats with a 1–6 Hz bandpass filter, are shown in the time domain and frequency domain.

At left, the occupant was attempting to breathe at 8 breaths per minute and was found to be breathing at 0.133 Hz, or 7.98 breaths/minute. Their heart rate recorded from a smart watch was 76 bpm at 0 min and 67 bpm at 5 min; the rate measured with the TruePODS was 1.266 Hz, or 76 bpm. At center, the occupant was attempting to breathe at 10 breaths per minute, and the recorded respiratory rate was 0.16 Hz, or 9.8 breaths/minute. Their heart rate recorded from a smart watch was 83 bpm at 0 min and 91 bpm at 5 min; the rate measured with the TruePODS was 1.40 Hz or 84 bpm. At right, the occupant was attempting to breathe at 10 breaths per minute, and their respiration was recorded at 0.16 Hz, or 9.6 breaths/min. Their heart rate recorded from a smart watch was 83 bpm at 0 min and 91 bpm at 5 min; the rate measured with the TruePODS was 1.44 Hz, or 86 bpm.

## Discussion

The extensive testing has provided data to validate high accuracy of occupancy/vacancy detection with the TruePODS in the most challenging scenario (stationary occupants vs empty room) and successful respiratory and heart rate detection for occupants in natural positions and orientations, with a single sensor, throughout a 3.4 × 8.5 m conference room. When the sensor was ceiling-mounted, presence was detected from occupants that were completely stationary (other than respiratory motion) in all locations in the room, including under the table, with accuracy of 93% using short 1–2 min windows. Respiratory rates were detectable in all room locations, and heart rate was detectable when the occupant sat at each seat at the conference table and four corners of the room. This is the first demonstration of a highly accurate occupancy/vacancy sensor that also provides physiological parameters.

This work shows greater than 93% detection of occupancy of largely stationary occupants by detecting breathing signals with Doppler radar. This approach provides a higher accuracy for stationary occupants than low-cost occupancy sensors based on passive infrared and ultrasound technology. Doppler radar sensors are straightforward to install, without concern for air vent location like ultrasonic sensors. Doppler radar sensors preserve privacy unlike camera-based sensors. Only Doppler radar technology can provide occupant vital signs in addition to occupancy data.

A limitation of Doppler radar for vital signs measurement is that motion from large or small body movements can obscure respiration and heart motion signals. This approach can, however, provide periodic snapshots of vital signs of occupants unobtrusively, without requiring their active participation. With additional algorithms, this sensor can be used to provide occupant vital signs over time, as well as occupant count^36^.

Another limitation of this work is that vital signs were only measured when the room had a single occupant. The authors have detected vital signs of multiple occupants in a room in other works^28^, and have plans to extend that work further. While addressing comfort of multiple occupants is a challenge, approaches include personal comfort systems such as desk fans, cooled chairs, footwarmers, and heated chairs^37,38,39^ that feed into HVAC predictive models^40^ to optimize both occupant comfort and energy efficiency.

Many occupants worry about privacy loss from ubiquitous sensors that measure occupancy and vital signs. The TruePODS sensor does not collect any personally identifiable information and cannot be used to link vital signs or locations to an individual.

During the COVID-19 pandemic, it became apparent that indoor environments are critical to wellness or illness. Because most people spend the vast majority of their time indoors, indoor environmental quality can have a significant impact on health. Air quality, lighting, temperature, humidity, water quality, and noise can all affect the well-being of a building’s occupants. While these parameters can be sensed directly, sensing the impact on humans has been challenging to date. Future work will include finding correlations between heart rates and respiratory rates and changes in these values with occupant wellness and comfort, so the vital signs can be used to proactively make positive changes in the environment to benefit occupants without requiring their active participation.

This work includes the first extensive data collection for a Doppler-radar based occupancy sensing and vital signs detection system in a realistic office environment. Hours of data with different occupants in different locations and positions were tested. Occupancy and vacancy were accurately differentiated over 93% of the time using both wall and ceiling-mounted configurations, in challenging circumstances where occupants were mostly stationary. Heart and respiratory rates were accurately detected in all seats in the conference room with a single ceiling-mounted sensor.

## Data Availability

The datasets generated and analyzed during this work and code developed in this work can be made available on reasonable request to the corresponding author.
